# ZnO-Embedded Carboxymethyl Cellulose Bioplastic Film Synthesized from Sugarcane Bagasse for Packaging Applications

**DOI:** 10.3390/polym17050579

**Published:** 2025-02-22

**Authors:** Anand Vyas, Sun-pui Ng, Tao Fu, Ifrah Anum

**Affiliations:** 1 Division of Science, Engineering and Health Studies, SPEED, The Hong Kong Polytechnic University, Hong Kong 999077, China; zerance.ng@cpce-polyu.edu.hk (S.-p.N.); ifrahanum@cpce-polyu.edu.hk (I.A.); 2 Key Laboratory of Biomedical Information Engineering of Ministry of Education, School of Life Science and Technology, Xi’an Jiaotong University, Xi’an 710049, China; taofu@xjtu.edu.cn

**Keywords:** carboxymethyl cellulose, ZnO nanoparticles, bioplastic, sustainable packaging

## Abstract

This research explores the synthesis of carboxymethyl cellulose (CMC) for the development of a cost-effective bioplastic film that can serve as a sustainable alternative to synthetic plastic. Replacing plastic packaging with CMC-based films offers a solution for mitigating environmental pollution, although the inherent hydrophilicity and low mechanical strength of CMC present significant challenges. To address these limitations, zinc oxide nanoparticles (ZnO NPs) were employed as a biocompatible and non-toxic reinforcement filler to improve CMC’s properties. A solution casting method which incorporated varying concentrations of ZnO NPs (0%, 5%, 10%, 15%, 20%, and 25%) into the CMC matrix allowed for the preparation of composite bioplastic films, the physicochemical properties of which were analyzed using scanning electron microscopy, Fourier transform infrared spectroscopy, and X-ray diffraction. The results revealed that the ZnO NPs were well-integrated into the CMC matrix, thereby improving the film’s crystallinity, with a significant shift from amorphousness to the crystalline phase. The uniform dispersion of ZnO NPs and the development of hydrogen bonding between ZnO and the CMC matrix resulted in enhanced mechanical properties, with the film CZ_20_ exhibiting the greatest tensile strength—15.12 ± 1.28 MPa. This film (CZ_20_) was primarily discussed and compared with the control film in additional comparison graphs. Thermal stability, assessed via thermogravimetric analysis, improved with an increasing percentage of ZnO Nps, while a substantial decrease in water vapor permeability and oil permeability coefficients was observed. In addition, such water-related properties as water contact angle, moisture content, and moisture absorption were also markedly improved. Furthermore, biodegradability studies demonstrated that the films decomposed by 71.43% to 100% within 7 days under ambient conditions when buried in soil. Thus, CMC-based eco-friendly composite films have the clear potential to become viable replacements for conventional plastics in the packaging industry.

## 1. Introduction

Packaging plays an important role in protecting products from chemical and physical damage, extending their shelf life, preventing contamination, and ensuring safe transportation [1,2]. A variety of synthetic plastics, including polystyrene, polypropylene, polyamide, polyester, and polyethylene, are widely employed in packaging due to their favorable properties—including high strength, lightweight composition, chemical inertness, and cost-effectiveness. However, these synthetic plastics rely heavily on petrochemical resources and present significant challenges, primarily due to their resistance to chemicals and their poor degradation. Currently, the excessive use of these nondegradable plastics risks the health of humans and the environment. Still, global plastic production reached approximately 367 million metric tons in 2020, and it is estimated that by 2050, it could rise to approximately 1.1 billion tons [3]. Alarmingly, roughly 36% of this production is linked to short-term or single-use plastics intended for packaging, which are frequently disposed of in landfills or released into the environment. Recycling these plastics remains challenging. A report from the U.S. Environmental Protection Agency stresses that only 7% of the plastic waste generated each year is recycled.

Over the past four decades, a highly promising strategy to address these challenges has been the development of bioplastics as a replacement for synthetic plastics. Bioplastics are considered fourth-generation packaging materials, offering a sustainable and environmentally friendly alternative [4]. These materials are biodegradable and renewable, being derived from naturally available sources such as proteins, lipids, and polysaccharides. Among those, cellulose, the major component of plant biomass, is the polysaccharide found most abundantly in nature. Indeed, cellulose is of significant interest due to its high availability, biodegradability, and remarkable physical properties, and the fact that it possesses a unique structure that can be modified into various derivatives that are useful for diverse applications. One notable derivative is carboxymethylcellulose (CMC), which incorporates carboxymethyl (-CH_2_COOH) and hydroxyl (-OH) functional groups into the cellulose chain. Carboxymethylcellulose is regarded as a highly promising raw material for making bioplastic films for packaging applications due to its high transparency, good film-forming capabilities, effective barrier properties against gases and lipids, and thermal stability. However, despite these advantages, the hydrophilic nature of neat CMC film limits the film’s tensile strength and makes it an inadequate water barrier. These limitations have initiated significant research focused on enhancing the properties of CMC-based films to render them suitable for use in packaging. Researchers have explored various modification techniques, including the incorporation of nanomaterials [5], co-polymerization [6], and reactions with crosslinking agents [7], to effectively address and overcome these challenges.

In recent years, the packaging industry has made considerable advancements by incorporating nanomaterials. Nanomaterials are materials with at least one dimension that measures less than 100 nm, and they are classified as nanoparticles (NPs), nanotubes, and nanolayers. A composite that incorporates NPs can produce high-performance and innovative materials. Nanoparticles are broadly categorized into two main types: *inorganic NPs*, such as metal oxide NPs, nano clays, and CNTs, and *organic NPs,* such as starch nanocrystals, lignocellulosic materials, and chitin whiskers [8]. Research indicates that inorganic nanoparticles, in particular, exhibit strong interfacial interactions in bioplastics. Various metals and metal oxide NPs, such as zinc oxide (ZnO) NPs [9], magnesium oxide NPs [10], titanium dioxide NPs [11], and silver NPs [12] have been investigated for their effects on bioplastic properties. Compared with other metal oxide NPs, ZnO is cost-effective and easy to handle, and its crystalline structure, ample surface area, and useful mechanical properties make it an ideal candidate for reinforcing bioplastic composites. Moreover, ZnO is water-insoluble, which can enhance the water resistance of bioplastic films. Furthermore, ZnO is recognized as a multifunctional filler, due to its impressive antimicrobial properties and the feature that when exposed to light, it can generate reactive oxygen species that can kill bacteria. Besides these properties, the biocompatibility and non-toxic nature of ZnO NPs have garnered considerable attention. It is crucial to address concerns regarding the potential migration of nanoparticles—particularly in food packaging—and their impact on human health. Studies indicate that ZnO NPs exhibit low toxicity and minimal migration under regulated conditions. The Food and Drug Administration (FDA) has approved ZnO as safe for human consumption, recognizing that trace amounts are even essential for human health [13,14]. Recent studies further support the feasibility of using ZnO NPs in bioplastic films for packaging applications. For example, incorporating ZnO NPs at various concentrations into chitosan films has been shown to enhance mechanical strength and antibacterial performance [13]. Another study demonstrated that the addition of ZnO nanoparticles along with cress seed markedly improved the physical, mechanical, and permeability properties of CMC-based films [15]. Similarly, composite films of Zein and gelatine loaded with ZnO NPs and polyphenols have been explored as a promising packaging material [16]. Collectively, these findings underscore the potential of ZnO Nps as versatile reinforcing agent for the development of scalable, eco-friendly, and sustainable bioplastic packaging solutions.

Therefore, in this work, we developed CMC-ZnO composite bioplastic films that exhibited good structural, mechanical, thermal, morphological, and barrier properties. We used sugarcane bagasse (SCB) as the raw material for the synthesis of CMC. SCB is a non-consumable waste material that is generated from the farming industry. The world’s annual production of sugarcane is approximately 1.6 billion tons, with roughly 280 kg of bagasse generated per ton. Remarkably, SCB possesses a significant cellulose content, ranging from 40% to 50%, but despite its cellulose-rich composition, most of the world’s bagasse remains unutilized [13]. Our approach aims not only to reduce the cost of CMC but also to contribute to agricultural waste management. Most of the existing research on CMC composites with metal oxide NPs has focused on commercially available CMC with low concentrations of NPs. Thus, our research seeks to expand the knowledge base and explore the potential for utilizing higher loadings of ZnO NPs in SCB-derived CMC bioplastic films. The resulting sustainable bioplastic films could then serve as functional, environmentally safe packaging materials.

## 2. Materials and Methods

### 2.1. Materials

Sugarcane bagasse was collected from a juice stall in Sham Shui Po, Hong Kong. Purchases of zinc oxide (ZnO, 50 nm), sodium hydroxide (NaOH, 98%), ethanol (C_2_H_5_OH, 99.7%), acetic acid (CH_3_COOH, 100%), sulfuric acid (H_2_SO_4_, 98%), sodium hypochlorite (NaOCl, 12%), hydrogen peroxide (H_2_O_2_, 35%), 2-propanol (C3H_8_O, 99.7%), and monochloroacetic acid (ClCH_2_CO_2_H, 99%) were bought from Orient Chemicals & Lab Supplies Co., Ltd. All purchases were analytical-grade chemicals that did not require any additional purification. Deionized (DI) water was used for making all of the solutions.

### 2.2. Composite Bioplastic Film

#### 2.2.1. Pre-Treatment of Sugarcane Bagasse

To enhance handling and processing efficiency, we reduced the size of the SCB to 1–3 cm and then dewaxed it by heating it in a 1:1 (*v*/*v*) mixture of ethanol and DI water at 60 °C for 1.5 h. This process was repeated to effectively wash away any impurities, such as wax, marrow, and dirt. After dewaxing the SCB, we thoroughly rinsed it with DI water and dried it at 70 °C. Finally, the dried SCB was pulverized to a fine powder that could pass through a 0.25 mm size screen.

#### 2.2.2. Isolation of the Cellulose

We extracted the cellulose from the SCB following a method outlined by a previous study, with some modifications [14]. The process involved a multi-step approach, starting with alkali hydrolysis followed by acid hydrolysis and a subsequent bleaching step to obtain pigment-free cellulose. The alkali hydrolysis process started by mixing 2% NaOH solution with 50 g of SCB powder (dry basis) in a beaker and then heating to 80 °C and maintaining that for 2 h to remove the lignin and hemicellulose from the SCB fibers. The resulting slurry was strained and the alkali-treated SCB fibers were subsequently washed using DI water. To eliminate any remaining lignin and other impurities, an acid hydrolysis step was performed, in which the fibers were stirred in a 4% (*v*/*v*) H_2_SO_4_ solution at 80 °C for 2 h. Following that treatment, the fibers were again washed until the pH reached 7. To achieve the desired degree of whiteness, the isolated fibers underwent a two-step bleaching process using 4% *v*/*v* of sodium hypochlorite and then hydrogen peroxide at 80 °C for 1 h. The bleached, pigment-free fibers were washed and dried overnight at 70 °C. Finally, these dried solid fibers were referred to as cellulose.

#### 2.2.3. Synthesis of Carboxymethyl Cellulose

The conversion process of cellulose to CMC was conducted following a two-phase approach, as discussed in previous studies [15]. In the initial phase, known as mercerization, cellulose was added to a mixture containing 400 mL of isopropanol and 100 mL of 30% (*w*/*v*) NaOH. The resulting mixture was subsequently incubated for 1 h and maintained at a temperature of 60 °C to facilitate alkalization of the cellulose. Later on, the cellulose fibers were transferred to the second phase, where they were subjected to etherification.

In this phase, the mercerized cellulose was stirred with a 400 mL 20% monochloroacetic acid (MCA) solution at 60 °C for 4 h. This allowed carboxyl groups to form on the cellulose backbone. When the reaction was complete, the sample was thoroughly filtered and washed using ethanol. The resulting product was identified as CMC, which was dried overnight at 60 °C for subsequent processing.

#### 2.2.4. Fabrication of CMC-ZnO Composite Bioplastic Film

Following previous studies, we employed a simple blending and solution casting method to prepare the CMC-ZnO composite bioplastic films [16,17]. We added 10 g of CMC (dry basis) to 100 mL of DI water to make a homogeneous solution. Zinc oxide NPs were homogenized separately in DI water at various loadings (5%, 10%, 15%, 20%, and 25% *w*/*w* of CMC) and were then added to the prepared CMC solution. To ensure good particle dispersion and prevent agglomeration, the solution was stirred continuously at 4000 rpm using a magnetometer. We added 10 mL of 25% citric acid solution dropwise to the mixture as a cross-linking agent. The solution was then heated at 110 °C until it reached a viscous consistency, after which we poured 85 mL of the solution into a 200 mm glass Petri dish and allowed it to dry naturally at room temperature for 4 h and then dried further for 18 h in an oven at 65 °C. All of the resulting films were designated as “CZn”, where n denoted the ZnO loading in 5% increments, from 0 to 25%. For our comparative analysis, the pure CMC film with ZnO, CZ_0_, served as the control film. Before characterization, all of the film samples were preconditioned at 23 °C and 50% relative humidity (RH) for 24 h.

### 2.3. Characterization and Performance Evaluation

#### 2.3.1. Thickness, Density, and Yield

**Figure 1:**
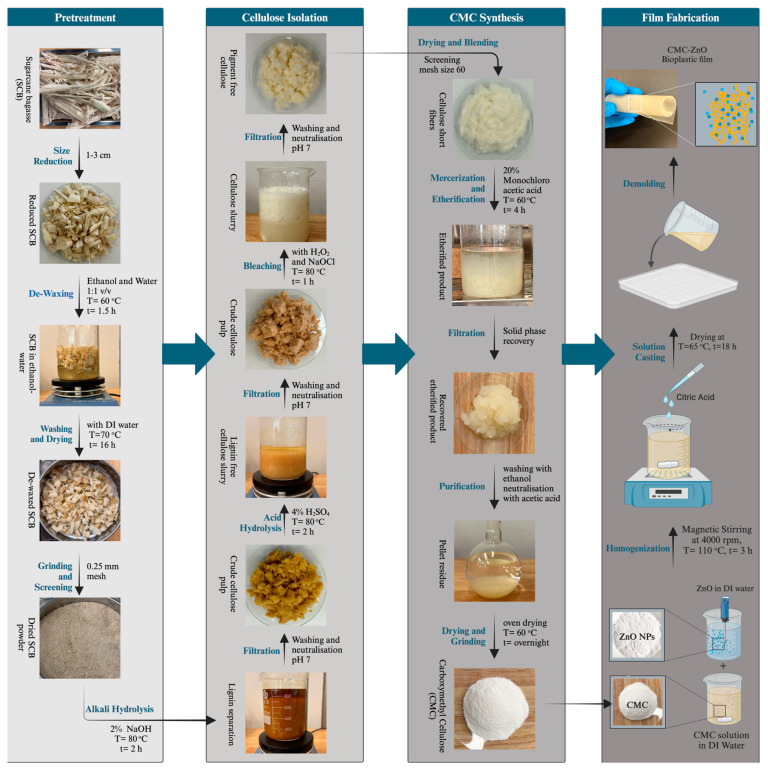
Schematic diagram illustrating the stages of CMC synthesis and the development of this study’s bioplastic film.

Thickness is a critical parameter that affects the performance of packaging films because it influences a film’s physical, mechanical, and overall functional properties. Figure 1 shows the step-by-step process of the fabrication of CMC-ZnO composite films. To ensure consistency, the thickness was controlled by adjusting the volume of the solution cast into Petri dishes of a fixed diameter. Thickness measurements were conducted following ASTM D6988 standards [18]. A Mitutoyo, Japan digital Vernier Caliper was employed to measure each film’s thickness at five different points, and the average value was recorded.

In addition to determining the film’s thickness, we calculated the film’s (g/cm^3^) density (ρ) using Equation (1):

ρ = [m/(A × L)],(1)

where “A” is the film’s area (in cm^2^), “L” is the film’s thickness (in cm), and “m” is the constant mass (in g) of the film after drying.

Similarly, we calculated the film’s nominal yield (*Yn*) or specific surface area using Equation (2):

*Yn* = [1/(ρ × L]. (2)

#### 2.3.2. Mechanical Properties

The mechanical properties of this study’s bioplastic films were assessed following the ASTM D882–18 standards [19] using the MTS Exceed model E42 Electromechanical Tester. Rectangular strips, with the dimensions of 11 cm × 3 cm, were carefully cut from the center of the films, and the mean thickness for each sample was considered for calculations. We set the initial grip separation at 60 mm and adjusted the crosshead speed to 28 mm/min. To ensure statistical significance, we tested each sample in triplicate and noted the mean value. Tensile strength was calculated by taking the maximum load that a sample could endure before failure and dividing it by its initial cross-sectional area, as shown in Equation (3):

σ (MPa) = F/A.(3)

Tensile elongation at break (EAB) was evaluated by measuring the percentage increase in the sample’s length until rupture, using Equation (4):

EAB (%) = [(L − L_0_)/L_0_] × 100. (4)

#### 2.3.3. FTIR Spectroscopy

FTIR spectroscopy was performed on the bioplastic films using a FTIR analyser (Vertex 70, Bruker, Berlin, Germany) to identify the functional groups and structure of the composite bioplastics. The films were cut precisely into (2 cm × 2 cm) samples and analyzed with the Attenuated Total Reflectance (ATR) method. The analysis included 64 scans over a wavelength range of 400–3500 cm⁻^1^, and a 4 cm⁻^1^ resolution. The spectral data obtained were then processed using OPUS software (version 7.5) for further analysis.

#### 2.3.4. X-Ray Diffractometry

The structural properties and crystallinity of the films were examined using a Smart Lab Rigaku X-ray Diffractometer. The instrument was operated at 40 kV and a 30 mA current, with data collection occurring at 2θ angles from 20° to 80°, with a scan rate of 0.050° per minute. Square samples measuring (1 cm × 1 cm) were analyzed using CuKα radiation in general focus PB mode.

#### 2.3.5. Scanning Electron Microscopy

Microstructural analysis of the surface of the synthesized bioplastic films was performed using a tabletop scanning electron microscope (TM4000 Plus Hitachi^®^, Tokyo, Japan). Before imaging, (1 cm × 1 cm) film samples were cut and coated with a thin metallic layer using a magnetron sputter (MSP-1S, Vacuum Device Inc., Tokyo, Japan). Analysis was carried out at a current intensity of 45 μA and an acceleration voltage of 15 kV while maintaining a working distance of 11.5 mm. The TM4000 software (version 1.5) was used to capture the microphotographs.

#### 2.3.6. Moisture Content and Moisture Absorption

Moisture content and moisture absorption refer, respectively, to the amount of moisture contained in the material and the moisture it takes up over time. These characteristics were determined according to the ASTM D4442-20 standard [20]. Pre-conditioned films were cut precisely into (2 cm × 2 cm) samples, and the initial mass, m_1_ (in g), was recorded. Subsequently, the samples were dried in a drying oven at 65 °C until a constant weight was reached. The mass after drying, m_2_ (in g), was noted. Then, the dried samples were placed in a closed chamber and kept there in storage for 24 h at 75% RH and 25 °C, after which they were reweighed to obtain m_3_ (in g). Each film sample was tested in triplicates under the same conditions and mean values were considered. The moisture content (MC) and moisture absorption (MA) were then calculated as percentages using Equations (5) and (6), respectively.

MC (%) = (m_2_ − m_1_)/m_1_, (5)

MA (%) = (m_3_ − m_2_)/m_2_. (6)

#### 2.3.7. Surface Wettability Using Contact Angle

The bioplastic films’ surface wettability or hydrophobicity was assessed by measuring their water contact angle (WCA) using a Sindatek goniometer model 100SB. For the measurements, (2 cm × 2 cm) samples of the film were cut and securely positioned on a stage of the analyser. A 5 μL droplet of DI water was then delivered to the film’s surface with the help of a micro syringe. After 5 s, images were captured and the WCA was determined using ImageJ software (version 1.54j). We repeated that process three times with each film, at different locations, to ensure consistency.

#### 2.3.8. Water Vapor Permeability

Using the gravimetric method, we evaluated the film’s water vapor permeability (WVP) in accord with ASTM E96-00 standards [21]. We cut circular film samples and sealed them onto 2 cm-diameter test tubes using silicon sealant. Each tube contained anhydrous calcium chloride, which maintained an internal relative humidity of approximately 0%. After recording the initial weight of the tube and film assembly, it was placed for 24 h in a desiccator that contained a saturated sodium chloride solution, which provided a controlled environment of 75% RH at 26 °C.

Water vapor transmission through the film was facilitated by the vapor pressure gradient between the two sides of the film, and subsequently the desiccant absorbed the water vapor. The weight gain of the assembly was measured, and then the WVP was calculated using Equation (7):

WVP = Q/t × 1/A × L/Ps (RH_1_ − RH_2_). (7)

In this equation, WVP is water vapor permeability (in gm^−1^ · day^−1^ · Pa^−1^), “Q/t” is the rate of mass gain (in g/d), “A” is the area of the bioplastic film (in m^2^), “L” is the bioplastic film’s average thickness (in m), and “P_S_” is the water vapor’s saturation pressure at tested temperature (in KPa), while “RH_1_” is the relative humidity inside the test tube and “RH_2_” is the relative humidity in the desiccator.

#### 2.3.9. Oil Permeability Coefficient

To measure the films’ oil permeability, the films were carefully sealed over the openings of test tubes containing 4 mL of cooking oil. The sealed test tubes were then inverted and placed on filter papers of known initial weight. Each assembly was incubated for 7 days, after which the filter paper’s weight was re-measured. The oil permeability coefficient, (P_0_), was calculated next using Equation (8):

P_0_ = (ΔQ × L)/(A × t), (8)

where P_0_ represents the oil permeability coefficient (in g · mm · m^−2^ · day^−1^), “ΔQ” is the change in the filter paper’s weight (g), “L” is the film’s thickness (in mm), “A” is the film’s area (in m^2^), and “t” is the incubation time (in days).

#### 2.3.10. Thermogravimetric Analysis

The thermal characteristics of the bioplastic films were evaluated using a thermal analysis system (TGA-DSC3+ Mettler Toledo Columbus USA). The experiments were conducted in a nitrogen atmosphere while maintaining a consistent flow rate of 50 mL/min. Between 2 and 3 mg of individual film samples were loaded in alumina crucibles and exposed to temperatures ranging from 25 to 700 °C, while maintaining a constant heating rate of 10 °C/min. We monitored the change in sample mass as a function of temperature. The data were acquired using the STARe software (version 14.00), and the resulting TGA thermograms were plotted and analyzed.

#### 2.3.11. Biodegradability

The degradation of the bioplastic films was evaluated by measuring their weight loss in soil using a conventional soil burial method. Organic gardening nutrient soil was prepared by sieving to ensure a uniform particle size. Film samples, cut into pieces (2 cm × 2 cm), were initially dried in an oven at 60 °C for 24 h to remove moisture, and their initial weights were recorded. Next, we buried these samples at a depth of 5 cm in plastic pots filled with prepared soil. The experiment was conducted for 7 days at ambient temperature, with soil moisture maintained by spraying 10 mL of deionized water daily. Samples were retrieved on days 3, 5, and 7, cleaned, dried, reweighed, and then reburied in the soil at the same depth. The weight loss was calculated to assess degradation using Equation (9):

BD (%) = [(m_i_ − m_n_)/m_i_] × 100, (9)

where BD (in %) is the percentage of degradation, “m_i_” (in g) represents the film’s initial film weight, and “m_n_” (in g) is the final weight after “n” days in the soil.

## 3. Results and Discussion

### 3.1. Physical and Mechanical Properties

#### 3.1.1. Thickness, Density, and Yield

The thickness of a packaging film is an important parameter that influences its mechanical properties and its barrier properties. Thickness primarily depends on the formulation of the film’s solid content and the conditions of the film’s preparation [22]. Table 1 lists the thickness, density, and yield of the bioplastic films prepared in this study. In the formulations, the concentration of CMC was kept constant, meaning that the variation in thickness was mainly influenced by the content of ZnO in the matrix. The control film, CZ_0_, exhibited the minimum thickness, at 203 ± 18 µm. In contrast, increasing the ZnO content resulted in a notable increase in film thickness, with a maximum value of 368 ± 26 µm observed in CZ_25_ at 25% ZnO NPs. Similar findings have been documented in previous research, which observed that the thicknesses of CMC [23], pectin/alginate [24], and corn starch [25] films increased with higher loadings of ZnO NPs.

Density provides valuable insights into a film’s compression and overall functionality. Generally, higher density indicates more compact structures. The density measurements of the films in this study varied significantly, ranging between 1.120 ± 0.085 and 1.466 ± 0.064 g/cm^3^. The control film, CZ_0_, demonstrated the lowest density, while a slight increase was observed in the CZ_5_ film after the addition of 5% ZnO. A more substantial increase in density was noted in the films containing 10% to 20% ZnO NPs. The higher density of the ZnO NPs, which appear to have filled the voids and holes within the CMC matrix and created a more compact and denser film structure, likely explains the enhanced density [26]. However, at the elevated ZnO concentration of 25% (in film CZ_25_), a reduction in density was observed. This could have been due to the uneven distribution of ZnO NPs throughout the CMC matrix at this higher level of loading.

The nominal yield or specific area of the films provided the area we obtained per unit of mass of the film. A continuous increase in the values of *Yn* was observed due to the increase in the thickness and density of the films.

#### 3.1.2. Mechanical Properties

Measuring bioplastic films’ mechanical properties is essential for determining their suitability as packaging materials. These properties influence the packaging’s integrity and ability to withstand external stresses during handling, storage, and transportation conditions [27].

**Figure 2:**
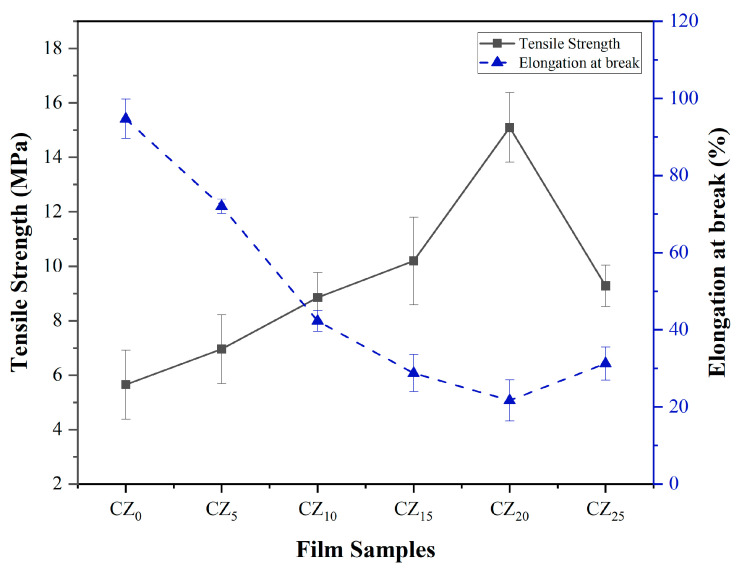
Effect of ZnO NPs on the mechanical properties of this study’s bioplastic films.

The tensile strength (TS) of the bioplastic films in this study was measured, and Figure 2 illustrates the results. The control film, CZ_0_, exhibited the lowest TS value, 5.65 ± 1.27 MPa. The incorporation of 5% ZnO NPs to CMC resulted in a slight increase in TS to 6.96 ± 2.26 MPa. As the concentrations of ZnO increased further, the films CZ_10_, CZ_15_, and CZ_20_ showed a continuous increase, with the highest value of 15.12 ± 1.28 MPa in the CZ_20_ film, which represents a 167% increase compared with the control film. This increase in TS may be ascribed to various factors: (a) hydrogen bond formation between adjacent Zn^2+^ of ZnO NPs and the negatively charged carboxyl group in the CMC polymer phase, (b) an excellent interfacial interaction causing the ZnO to be homogeneously dispersed, and (c) ZnO NPs filling in the gaps in the CMC chains and ultimately creating a tighter structure that improved the films’ performance significantly. Previous studies have reported similar results, in which the addition of ZnO NPs to CMC- [17,28] and chitosan-based films [29,30] resulted in increased TS. However, in the present study, with an increase in the ZnO concentration to 25%, the TS of film CZ_25_ decreased to 9.28 ± 0.77 MPa. The accumulation of ZnO NPs may have caused this reduction by creating non-homogeneity and weak areas within the film, as confirmed by the SEM image shown later in Section 3.2.3. Earlier studies had reported similar findings, in which the agglomeration of ZnO at higher contents caused starch bioplastic films to undergo a decrease in their tensile strength [9].

Tensile elongation at break (EAB) signifies the material’s stretchability or flexibility, and it is typically inversely related to TS [27]. The flexibility requirements of packaging films vary on the basis of the application. For instance, rigid packaging may need a lower EAB, whereas the wrap film requires a higher EAB. This study’s results indicated a significant reduction in the film’s EAB after adding ZnO, compared with the control film, likely because the ZnO increased the film’s rigidity, which hindered the deformation and movement of polymer chains in the matrix, hence reducing the ductility. Interestingly, an increase in EAB value was observed in the CZ_25_ film. These findings on EAB align with previous studies on chitosan/sodium alginate [31] and CMC [23] bioplastic films with ZnO NPs.

### 3.2. Characterization of Internal Structure

#### 3.2.1. FTIR Analysis

**Figure 3:**
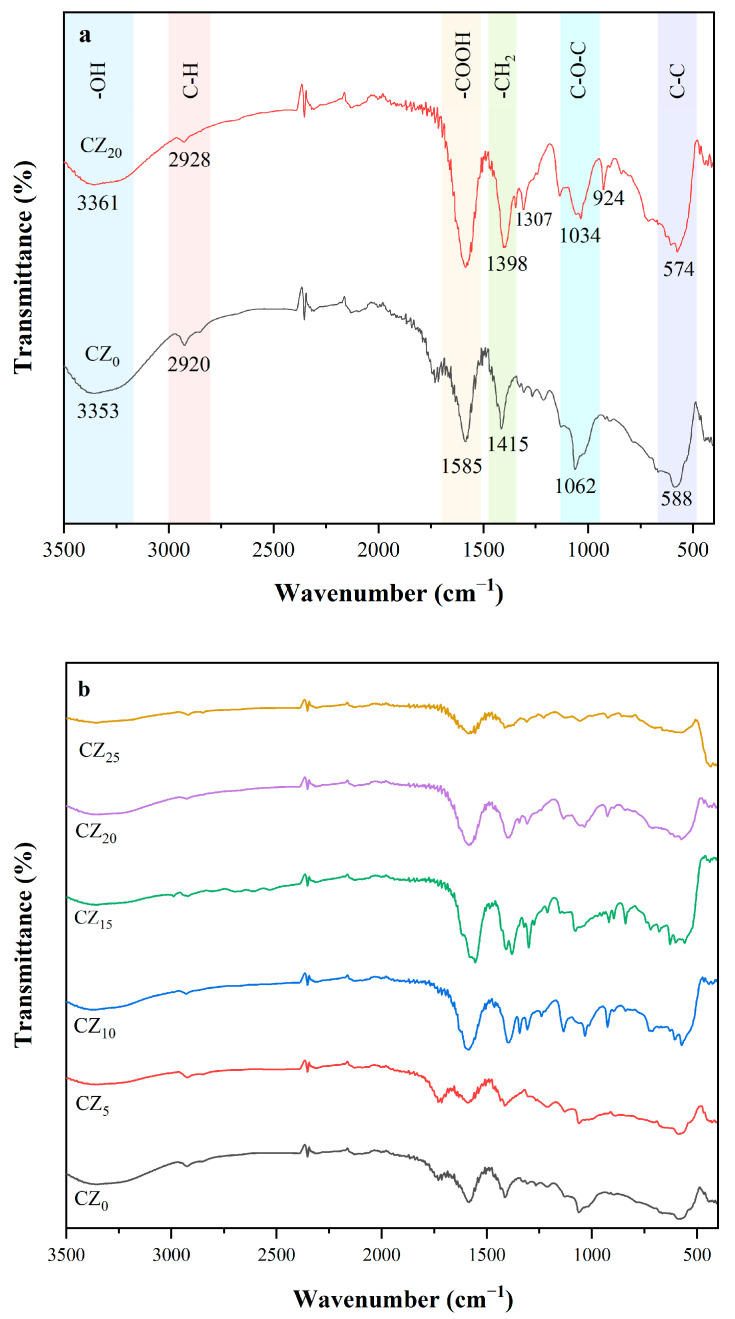
(**a**) Comparison of the FTIR spectra of CZ_0_ and CZ_20_. (**b**) The FTIR spectra of this study’s bioplastic films.

We investigated the CMC’s and ZnO NPs’ chemical functionalities and the potential interactions between the two by performing Fourier transform infrared spectroscopy. The spectra for both the control and the composite bioplastic films are presented in Figure 3a,b. In the spectrum of the pure CMC film (CZ_0_), a broad absorption band marker at 3353 cm⁻^1^ coincided with the stretching vibration of a hydroxyl group, while a band at 2920 cm⁻^1^ was associated with C-H stretching vibrations. Notably, in the fingerprint region, a prominent absorption band at 1585 cm⁻^1^ confirmed the stretching vibration of the –COOH group, and a second prominent absorption band, at 1415 cm⁻^1^, confirmed the scissoring vibrations of –CH_2_.

The band around 1062 cm^−1^ was assigned to C-O-C bond stretching vibrations [14,32]. These observed bands confirmed the presence of major carboxymethyl groups and indicated the successful formation of CMC from the sugarcane bagasse.

When the ZnO had been incorporated, the CMC and ZnO NPs interacted in ways that resulted in notable changes in the absorption frequencies observed in the CZ_20_ spectrum (Figure 3a). The shift and broadening of the –OH stretching band were attributed to the formation of new hydrogen bonds between ZnO and CMC [16]. The intensity of the 2920 cm^−1^ band was linked to decreasing asymmetric stretching in the –CH_2_ group in response to the introduction of the ZnO and also to increases in its concentration. In addition, the incorporation of ZnO NPs caused some of the peaks to shift to lower wavenumbers. For instance, the peaks that emerged at 1415 and 1062 cm^−1^ were shifted to 1398 and 1034 cm^−1^, respectively. Moreover, when the ZnO was added to the CMC, new absorption peaks appeared between 400 and 1400 cm^−1^ that likely were due to ZnO vibration. These shifts and changes in peak intensity suggest that an interaction occurring between the CMC and ZnO enhanced the compatibility between the CMC matrix and the ZnO and ultimately led to improvements in the composite films’ mechanical properties and water resistance.

#### 3.2.2. XRD Analysis

**Figure 4:**
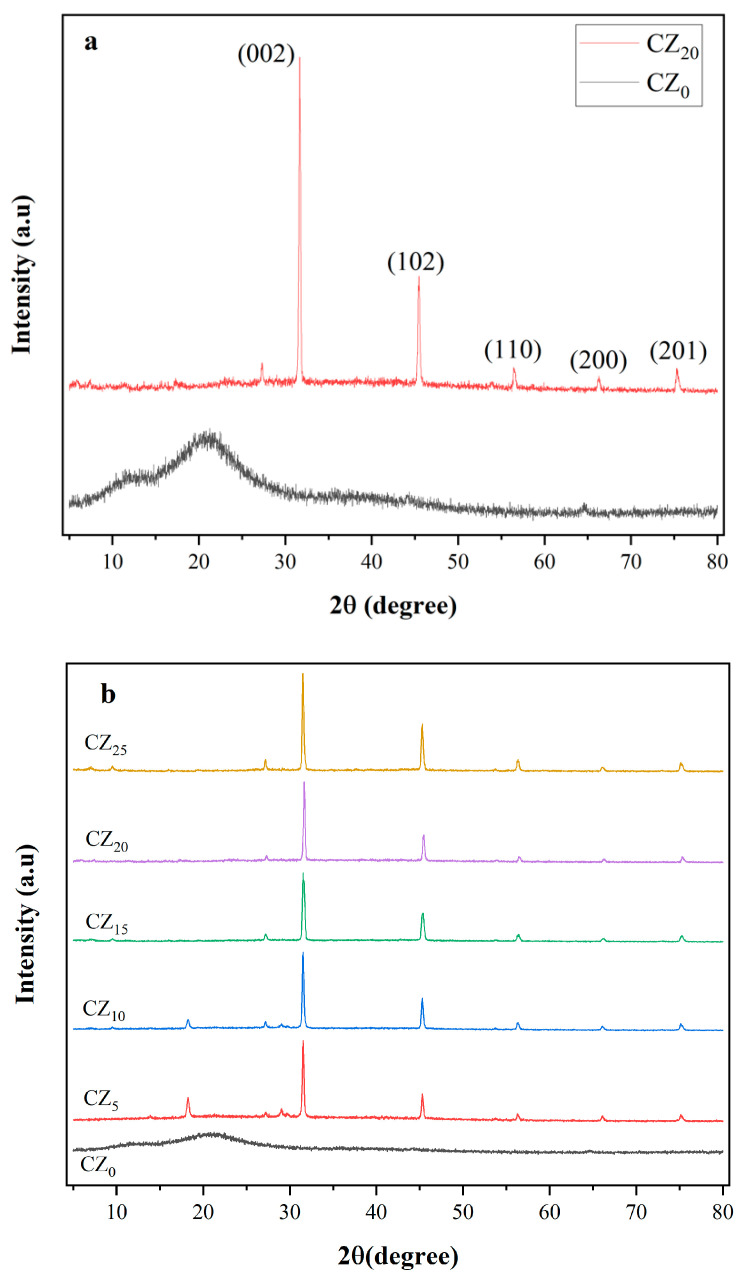
(**a**) Comparison of the XRD spectra of CZ_0_ and CZ_20_. (**b**) The XRD spectra of all of the bioplastic film samples.

X-ray diffraction analysis elucidated the crystallographic structure and phase composition of the bioplastic films, and the diffraction patterns of the control and composite bioplastic films are illustrated in Figure 4a,b. The diffractogram of the control film, CZ_0_, showed no visible strong peaks, but a broad and low-intensity peak were observed at approximately 2θ = 20°, indicating the amorphous or semi-crystalline nature of CMC [15]. This is characteristic of polymers, in which the molecular chains are arranged in a less ordered fashion. Another study had previously observed a similar diffraction spectrum in a CMC matrix synthesized from sugarcane bagasse [14]. In contrast, the CMC-ZnO composite bioplastic film exhibited sharper and more distinct peaks at 2θ = 31.5°, 45.3°, 56.2°, 66.1°, and 75.3°, corresponding, respectively, to the (002), (102), (110), (200), and (201) planes of wurtzite ZnO (JCPDS card No. 36–1451) [33,34]. These peaks represent the crystalline phases of ZnO, thus indicating the successful incorporation of ZnO NPs into the CMC matrix. The presence of distinct diffraction peaks suggests that the composite film possesses improved crystallinity compared with that in the pure CMC film. However, the absence of two peaks, when compared with the JCPDS card No. 36–1451, might be due to the effective dispersion of ZnO within the CMC matrix, as well as to the formation of their bonding, as confirmed by FTIR analysis. With increasing concentrations of ZnO NPs, the major diffraction peaks became increasingly pronounced and strong, indicating the formation of a highly crystalline structure. Moreover, the distinct CMC peak at approximately 2θ = 20° diminished with the concentration of ZnO in the composite. No peak shifts were detected, thus suggesting phase retention of ZnO. Overall, the addition of nanoparticles into the CMC increased the film’s crystallinity. As was the case with our results, the transition from amorphousness to the crystalline phase of bioplastic films by incorporation of ZnO was previously documented in cellulose- [35], starch- [36], and carboxymethyl chitosan/gelatin-based composite films [37].

#### 3.2.3. Surface Morphology

**Figure 5:**
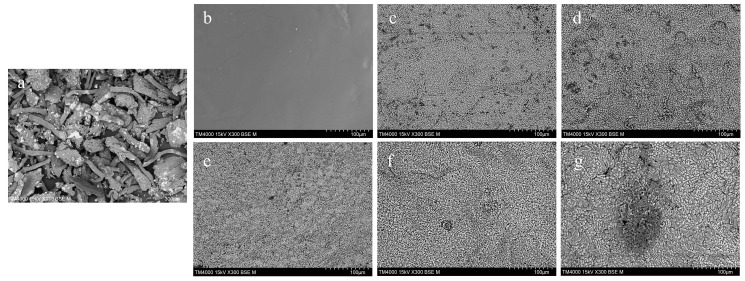
SEM images of the bioplastic films, at a surface view, of (**a**) synthesized CMC powder, (**b**) CZ_0_, (**c**) CZ_5_, (**d**) CZ_10_, (**e**) CZ_15_, (**f**) CZ_20_, and (**g**) CZ_25_.

The microstructural properties of a bioplastic film directly influence its mechanical, thermal, and barrier performance—properties that are influenced by the film preparation method, its composition, and the dispersion of NPs within the matrix. Understanding a film’s morphology is therefore essential to optimizing the concentration of NPs in film formulation. We examined the surface morphology of this study’s CMC and composite bioplastic films using SEM, with the resulting micrographs presented in Figure 5. The structure of synthesized CMC powder from SCB is depicted in Figure 5a. The morphology of the control film, CZ_0_, appeared clear, smooth, and compact, and the surface was observed to be free from bubbles and holes, as shown in Figure 5b. When the ZnO NPs had been added to the CMC, we observed a noticeable morphological change in the film surface, which we attributed to the interaction between the CMC and the ZnO NPs. The composite films exhibited a sharper morphology and heterogeneous surfaces Figure 5c–g. The ZnO NPs were evenly embedded throughout the composite, which suggests that they effectively prevented aggregation and contributed to the formation of crystalline structures. However, as is seen in Figure 5g, a degree of nonuniformity was observed in the film CZ_25_, thus indicating potential challenges to maintaining a consistent morphology at higher loadings of NPs.

### 3.3. Water Resistance and Hydrophobicity

#### 3.3.1. Moisture Content

Moisture is an inherent component of most films and coatings, primarily due to the use of water as a solvent in their production. Although water’s plasticizing effect significantly enhances the flexibility of biopolymer-based films, the elevated moisture levels can adversely affect the films, making them unsuitable for the packaging of moisture-sensitive items. Composite films that include hydrocolloids, such as CMC, are particularly prone to higher water retention because of their inherently hydrophilic characteristics. However, the inclusion of constituents such as emollients, essential oils, lipids, and nanoparticles can modulate the moisture content in composite bioplastic films [38]. Table 1 presents data on the moisture content of bioplastic films as a function of the ZnO NPs being at increasing concentrations. In this study, the control film, CZ_0_, had the highest values of moisture content (23.12 ± 0.85%), thus affirming the hydrophilicity of CMC. The measurements further indicate that adding ZnO NPs substantially reduced the amount of moisture in the prepared bioplastic films. Initially, the addition of 5% ZnO, in film CZ_5_, resulted in a 30.93% reduction in moisture, compared with our control film. As the ZnO concentration was increased, the values continued to drop and ultimately reached the lowest moisture content value, 10.38 ± 1.71%, in the CZ_20_ film, representing a 55.10% reduction compared with the control film. However, when the ZnO amount was raised to 25%, an increase in the film’s moisture content was observed, likely due to non-homogeneity and clustering of the nanoparticles creating free cavities in which the water molecules could accumulate [39].

#### 3.3.2. Moisture Absorption

The high moisture absorption of bioplastic films notably distinguishes them from synthetic plastics and greatly limits the extent of their applications. Fortuitously, incorporating metal nanoparticles has proven effective in reducing the moisture uptake of these bioplastic films [23]. Table 1 summarizes the results of our moisture absorption analysis on the fabricated films over 24 h, and as expected, the control film, CZ_0_, exhibited the highest percentage of moisture absorption (25.01%). Previous research has also underscored the high capability of CMC film to absorb moisture because of its hydrophilic properties [30]. Notably, in our results the moisture absorption decreased significantly upon the addition of ZnO NPs and with increasing concentrations of ZnO, with the films at 5%,10%, 15%, 20%, and 25% ZnO displaying respective moisture absorption levels of 18.97%, 18.12%, 17.71%, 14.04%, and 13.46%. This reduced absorption of moisture likely resulted from the robust bonding formed between CMC and ZnO, which limits the availability of free –OH groups and decreases the number of water-reactive sites in the films. Consequently, the resulting films have a less hygroscopic film matrix. In addition, ZnO NPs may reduce the number of open spaces available for moisture absorption, creating a zigzag path that impedes water molecules’ movements. Similar findings have been observed by researchers in studies on CMC-GNPs [40], CMC-TiO_2_ [26], and pectin-ZnO bioplastic films [41,42].

#### 3.3.3. Surface Wettability

**Figure 6:**
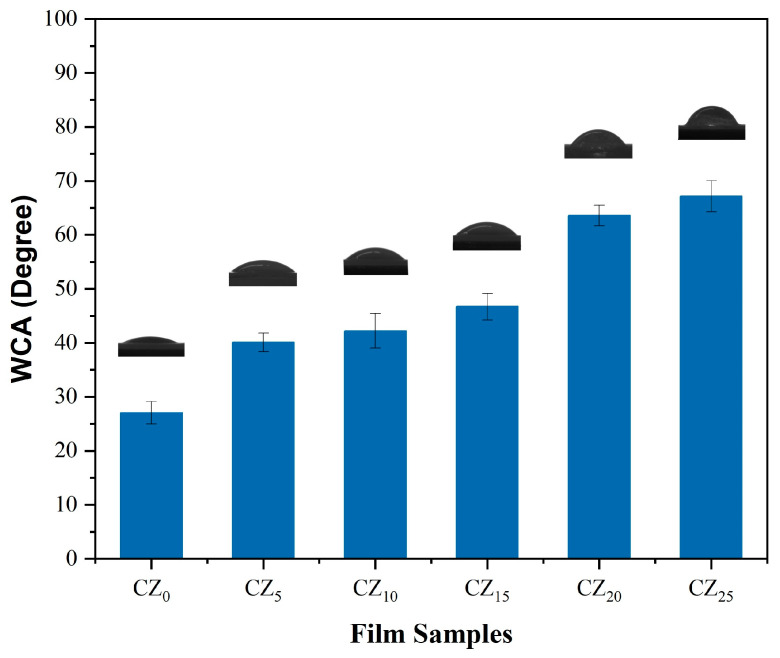
Water contact angles on this study’s bioplastic film surfaces.

An understanding of surface wettability is crucial for informing the design and development of packaging materials, because surface wettability influences a film’s capacity to repel or absorb water and other liquids. Research indicates a variety of wettability values for bioplastic films used in packaging. Some studies have reported films with contact angles below 90°, while others have created hydrophobic films with angles exceeding 90°. The selection between hydrophobic and hydrophilic packaging films is determined by the product’s specific requirements—for example, hydrophobic films repel water and are ideal for items that must remain dry and crisp, such as crackers and cookies. In contrast, hydrophilic films absorb moisture, which is beneficial for packaging products that need effective moisture management, such as certain fresh fruits and vegetables that release water vapor. The water contact angles of this study’s bioplastic films are illustrated in Figure 6. The control film, CZ_0_, showed the lowest water contact angle—27.06°—indicating a hydrophilic surface resulting from the CMC polymer backbone’s abundance of –OH groups. These hydroxyl groups and water molecules readily interact, leading to a low contact angle. The films’ WCA values were significantly increased with the addition of ZnO Nps, and the increment was dependent upon the concentration of NPs. Notably, a significant increase in the water contact angle was observed as the ZnO concentration increased from 15% (CZ_15_) to 20% (CZ_20_). This substantial increase can be attributed to a threshold effect in nanoparticle dispersion and surface migration. At lower ZnO concentrations, the nanoparticles remain embedded within the CMC matrix, whereas at higher loading, a proportion of ZnO NPs likely migrate to the film’s surface. This higher surface concentration not only reduces the surface free energy but may also alter the surface morphology by introducing micro or nanoscale roughness. According to the Cassie–Baxter model, such roughness can trap air pockets reducing the contact area between the water droplet and the film. This effect enhances hydrophobicity, contributing to the observed increase in the water contact angle. The highest WCA, 67.15°, was observed on the CZ_25_ composite film’s surface. This increased hydrophobicity results from the combined effect of film’s reduced surface free energy, the inherent insolubility of ZnO NPs in an aqueous solution [43] and their potential bonding with OH groups of CMC, as discussed earlier. These observations align with previous findings on the incremental changes in WCA values with the addition of ZnO NPs in CMC-, starch-, and chitosan-based bioplastic films [43,44,45,46].

### 3.4. Water and Oil Barrier Properties

#### 3.4.1. Water Vapor Permeability

In the research field on packaging materials, the study of films’ water vapor permeability is essential for developing materials that effectively inhibit water vapor diffusion, thereby enhancing the films’ ability to preserve and protect packaged contents from environmental factors. Water vapor permeability is significantly influenced by factors such as the film’s thickness, hydrophilicity, crystallinity, pore bending, and porosity. Incorporation of nanoparticles into packaging films can boost the films’ resistance against moisture transfer without compromising other key properties. In this study, as can be seen in Table 2, the pure CMC film, CZ_0_, showed the highest WVP value: 5.62 × 10^−6^ gm^−1^ · day^−1^ · Pa^−1^, and with increases in the concentration of ZnO Nps in the CMC matrix, the films’ values for of WVP gradually decreased. This reduction in WVP can be ascribed to several aspects of the films: (a) ZnO Nps filled empty spaces in the CMC matrix and created zigzag and polygonal paths that impeded and prolonged the movement of water molecules, (b) the bonding between ZnO NPs and the CMC reduced the availability of free hydroxyl groups, thus lowering hydrophilicity, (c) ZnO crystal nuclei formation in the films decreased the density of pores and increased their curvature, and (d) ZnO NPs exhibited lower hydrophilicity than pure CMC does. Indeed, the literature has reported significant variations in the measured values of WVP of CMC-based bioplastic films. Our findings align with previous studies that showed a reduction in WVP upon the incorporation of various metal oxides [17,23]. However, our results contradict earlier research that observed an increase in the WVP values of CMC/gelatine films when the ZnO NPs were added—a phenomenon that was linked to increased porosity [47].

#### 3.4.2. Oil Permeability

Oil permeability (OP) is another important property of film packaging, particularly in the food packaging industry, because it directly affects the barrier that prevents oil and fat migration. High oil permeability can cause contamination, spoilage, and reduced shelf life, and the oil permeability of a film is influenced by the film material’s number of lipophilic groups [48]. Previous studies have indicated that CMC-based composite films possess effective oxygen and lipid barrier properties [49]. In the present study, while the composite film’s concentration of CMC was constant, the variation in the oil resistance properties of the film was studied solely as the function of ZnO. The oil permeability coefficients of the control and composited film samples were measured over a period of 7 days at ambient temperature. Daily observations revealed no signs of oil transfer through the films until Day 6 (144 h). However, on Day 7, faint traces of oil were found on the filter paper beneath the films CZ_0_, CZ_5_, CZ_10,_ and CZ_15_, whereas no oil permeability was detected in the CZ_20_ and CZ_25_ bioplastic films throughout the experiment. These findings suggest that the CMC film itself was a good oil repellent. Moreover, the higher content levels of ZnO not only prolonged the films’ time until oil penetration but also reduced their oil permeation coefficients. The ZnO NPs enhanced the films’ degree of crystallization (Figure 4) and decreased their free volume, thus creating a more tortuous pathway for oil molecules to travel and thereby improving oil barrier performance. A previous report demonstrated that increases in the concentration of ZnO in the ZnO-PLA coating on multilayer packaging extended the packaging’s oil permeation time [50].

### 3.5. Thermal Stability

**Figure 7:**
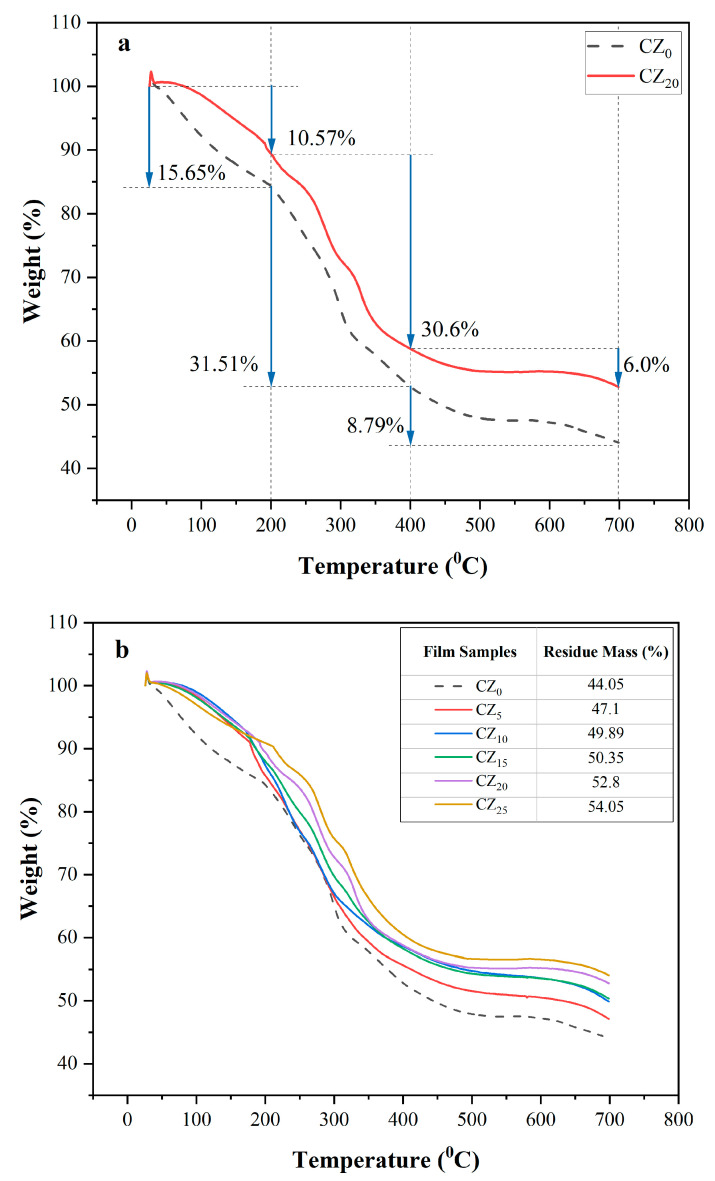
(**a**) Comparison of the TGA thermograms of CZ_0_ and CZ_20_; (**b**) thermograms of all of this study’s bioplastic film samples.

Thermogravimetric analysis (TGA) is employed to assess the thermal and oxidative stability of bioplastic films, particularly at elevated temperatures—properties that are important for packaging films which may encounter high temperatures during processes such as sealing [51]. Thermogravimetric analysis also allows for the evaluation of the effects of various additives and processing conditions on polymeric degradation. It is widely believed that the addition of nanoparticles into polymeric materials can significantly improve their thermal stabilities [52]. We conducted TGA on both control and composite bioplastic films, and the resulting thermograms are presented in Figure 7a,b. All of the thermograms revealed a three-stage weight-loss pattern for all of the samples. In the first stage, within the range of 25 to 200 °C, the weight reduction was primarily due to the evaporation of water molecules and unknown volatile organic matter within the films [53]. Notably, the control film, CZ_0_, exhibited the most significant weight loss—approximately 15.65%—while the composite films demonstrated comparatively smaller weight losses within the same temperature range. For instance, the film CZ_20_ showed a 10.57% weight loss. This discrepancy was linked to the low moisture content in the composite films, and also to the compact structure of CMC-ZnO, which hindered the evaporation of water molecules. This observation is further corroborated by the moisture content data presented in Table 1, which indicated that the film CZ_0_ had the highest amount of MC (%), and that value decreased continuously with the inclusion of increasing amounts of ZnO Nps in the composite films.

During the second stage of weight loss, between 200 and 400 °C, the loss in mass was linked to the structural degradation of monomers and functional groups in the polymer backbone [54,55]. A slightly smaller amount of weight loss in composite films was observed in this range compared with that of CZ_0_, and that finding was linked to the strong hydrogen bond interactions of the CMC matrix with the ZnO NPs. In addition, the high thermal stability of ZnO enables it to function as a thermal insulator and a barrier against the mass loss of volatile matter generated during thermal decomposition [56]. The third phase of weight loss, occurring between 400 and 700 °C, likely resulted from the complete decomposition of residual carbon [32]. At 700 °C, we observed a noticeable difference in the residual mass of the films, with the films that contained a higher content of ZnO retaining more mass Figure 7b. Moreover, as the ZnO concentrations were increasing, the films’ degradation curves shifted to a higher temperature than was the case with the control film, CZ_0_, thus indicating improved thermal stability.

### 3.6. Biodegradation Study

Biodegradability evaluates the environmentally friendly nature of bioplastic films and offers a solution to non-degradable synthetic plastics. Biodegradability measures the rate at which bioplastic film decomposes in the soil, and it is influenced by factors such as microbial activity, soil moisture level, atmospheric conditions, and film composition [57,58]. The literature indicates that the degradation rate of bioplastic films containing nanoparticles shows varied trends, exhibiting both slower, and, in some cases, faster rates of degradation.

**Figure 8:**
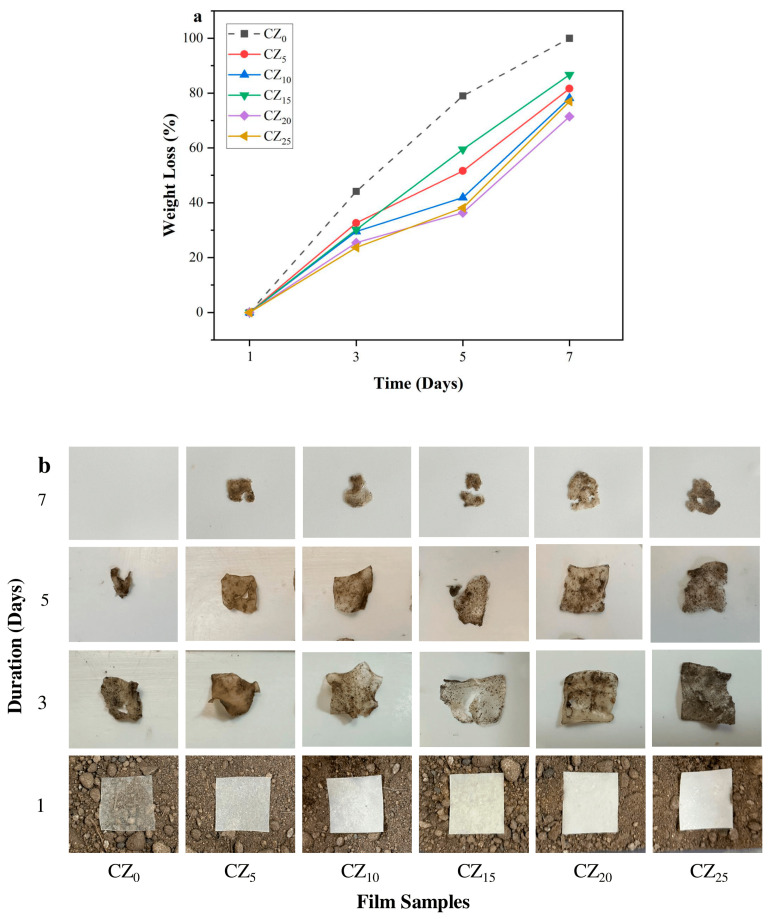
(**a**) Percentage of degradation of this study’s bioplastic films; (**b**) photographic visuals of the degradation process on days 0, 3, 5, and 7.

The results of our biodegradability test are depicted in Figure 8. Where Figure 8a illustrates the weight loss profile of the bioplastic films, Figure 8b contains the visual images of films after recovering from burial in the soil over a period of 7 days. The films exhibited significant weight loss during the initial 3 days, and then the rate gradually decreased from day 3 to 5. In the beginning of the test, the film’s surface was fully exposed to microorganisms that colonized the film and used it as a source of carbon, thereby accelerating the degradation process. As degradation progressed, the availability of easily degradable components decreased, leaving behind more complex compounds that were less susceptible to breakdown. Moreover, the reduced surface area of the remaining film limited microbial access, leading to a slower degradation rate with time [59]. The pure CMC bioplastic film, CZ_0_, demonstrated the highest weight loss, achieving 44.12, 78.95, and 100% weight loss on days 3,5, and 7, respectively. This high degradation was linked to the abundance of –OH groups in the CMC that facilitated hydrolysis by absorbing water from the soil. The water absorption led to thickening, further promoting the degradation process [9]. In contrast, the introduction of ZnO NPs into the CMC matrix led to a notable decrease in weight loss in the composite films, and notably, the films’ degradation rates were reduced further by the increasing concentrations of ZnO. For instance, the percentage weight loss in the composite films CZ_5_ through CZ_25_ ranged, respectively, from 32.61 to 23.64% by day 3. As the soil burial time progressed, the films continued to degrade, with the final weight loss values on day 7 recorded as 81.62 (CZ_5_), 78.12 (CZ_10_), 86.67 (CZ_15_), 71.43 (CZ_20_), and 76.92% (CZ_25_). The film CZ_20_ exhibited 28.57% less weight loss compared with the control film, due to the impact of ZnO. The presence of ZnO NPs creates a denser and more compact film structure, as previously discussed in WVP and OP studies. This denser structure acts as a physical barrier, restricting soil microorganisms from accessing the CMC matrix. In addition, ZnO NPs are insoluble and exhibit good hydrophobic properties, as evidenced by our moisture-related studies and water contact angle measurements. Consequently, the films become less moist, creating an environment that is less conducive to microbial degradation. Furthermore, ZnO NPs have remarkable antimicrobial properties that can suppress the growth and function of microbes involved in CMC degradation, thereby decreasing the overall degradation rate. Similar findings have also been presented by other researchers, in starch-ZnO composite films, thus indicating that increased ZnO loading can prolong the biodegradation time [9].

## 4. Industrial Scalability

While the lab-scale synthesis and characterization of CMC-ZnO bioplastic films has been successfully demonstrated, several factors must be addressed to facilitate industrial-scale production of these films. Our primary feedstock, sugarcane bagasse, is an abundant agricultural waste; however, its availability can be influenced by regional variability and seasonal fluctuations, potentially affecting the consistency of CMC production. Effective inventory management strategies could help mitigate this issue. Scaling up the cellulose extraction process will require transitioning from batch processing to continuous or semi-continuous reactor systems. Such systems, with enhanced mixing, improved heat transfer, and precise pH control, can significantly improve reaction uniformity. Moreover, advanced extraction techniques—such as ultrasound or microwave assisted methods—could reduce reaction times and lower energy consumption. Optimizing chemical usage is also crucial for large-scale operations. By recovering and reusing reagents such as NaOH, H_2_SO_4_, and bleaching agents we can improve both the economic and environmental sustainability at large-scale operations. In terms of film fabrication, solution casting is the most extensively studied method. However, it can be energy intensive and may lead to prolonged drying times and film inhomogeneities, when applied at larger volumes. In contrast, alternative methods such as extrusion and molding could offer higher productivity and efficiency, making them more suitable for industrial-scale production. By addressing these factors, SCB-derived films can be produced on an industrial scale as a promising sustainable packaging solution.

## 5. Conclusions

This study highlights the effective use of sugarcane bagasse, an agricultural waste product, for synthesizing carboxymethyl cellulose (CMC). Bioplastic films were manufactured by incorporating various concentrations of ZnO NPs as reinforcing filler into the CMC matrix, utilizing the solvent casting technique. Characterization through SEM, X-ray diffraction, and FTIR spectroscopy confirmed the formation of hydrogen bonding interactions between the CMC chains and the ZnO NPs. These analyses also revealed uniform dispersion of ZnO NPs up to a concentration of 20%. The inclusion of ZnO NPs significantly enhanced the crystallinity of the composite films, and that led to improved thermal stability and mechanical properties. Notably, the bioplastic film with 20% ZnO (CZ_20_) achieved a remarkable tensile strength of 15.12 ± 1.28 MPa, thus showing a substantial 167% increase in strength compared with the control film. This study further assessed the moisture-related properties and barrier characteristics of the bioplastic films. Results indicated that higher concentrations of ZnO NPs contributed to denser, more compact films, thereby reducing both moisture content and moisture uptake. Water vapor permeability was significantly reduced—by 24.5%—reaching a minimum of 4.24 × 10⁻⁶ gm⁻^1^ · day⁻^1^ · Pa⁻^1^. Moreover, the incorporation of ZnO also decreased both the oil permeability coefficient and the permeation time, with films CZ_20_ and CZ_25_ showing no oil permeability over 7 days, thus suggesting their suitability for packaging applications. Furthermore, water contact angle measurements demonstrated that ZnO NPs effectively reduced the hydrophilicity of CMC, indicating a lower affinity of the film surface for water. Importantly, the bioplastic films exhibited excellent biodegradability, with 71.43% to 100% of their mass decomposing in soil within 7 days at ambient conditions. This rapid degradation ensures that the films are environmentally friendly and addresses growing concerns about conventional plastic pollution. Moreover, the use of agricultural waste as a basic material for CMC synthesis, together with a facile film fabrication process, offers economic feasibility for developing sugarcane bagasse as a synthesizing agent for sustainable packaging materials.

## Figures and Tables

**Table 1:** Physical and moisture-related properties of the bioplastic films in this study at various concentrations of ZnO ^1^.

Film Codes	Thickness µm	Density g/cm^3^	Yield cm^2^/g	Moisture Content (%)	Moisture Uptake (%)
CZ_0_	203 ± 18	1.120 ± 0.085	43.56 ± 2.32	23.12 ± 0.85	25.01 ± 0.17
CZ_5_	290 ± 08	1.264 ± 0.081	27.29 ± 3.50	15.97 ± 1.23	18.97 ± 1.72
CZ_10_	298 ± 17	1.544 ± 0.108	21.77 ± 4.65	13.08 ± 1.58	18.12 ± 0.005
CZ_15_	323 ± 40	1.502 ± 0.104	20.64 ± 2.38	11.26 ± 3.39	17.71 ± 0.16
CZ_20_	358 ± 21	1.562 ± 0.063	17.91 ± 1.30	10.38 ± 1.71	14.04 ± 0.79
CZ_25_	368 ± 26	1.466 ± 0.064	18.56 ± 2.24	14.74 ± 1.64	13.46 ± 1.63

^1^ Values represent the mean ± standard deviation of triplicates.

**Table 2:** Water vapor permeability (WVP) and oil permeability (OP) of this study’s bioplastic films at various concentrations of ZnO ^1^.

Film Codes	WVP gm^−1^ · day^−1^ · Pa^−1^	OP g·mm · m^−2^ · day^−1^
CZ_0_	5.62 × 10^−6^	37.31
CZ_5_	5.02 × 10^−6^	32.76
CZ_10_	4.80 × 10^−6^	26.21
CZ_15_	4.58 × 10^−6^	13.83
CZ_20_	4.24 × 10^−6^	x
CZ_25_	4.29 × 10^−6^	x

^1^ Values represent the mean ± standard deviation of triplicates.

## Data Availability

Data are contained within the article.
